# Microbial "social networks"

**DOI:** 10.1186/1471-2164-16-S11-S6

**Published:** 2015-11-10

**Authors:** Mitch Fernandez, Juan D Riveros, Michael Campos, Kalai Mathee, Giri Narasimhan

**Affiliations:** 1 Bioinformatics Research Group (BioRG), School of Computing and Information Sciences, and Biomolecular Sciences Institute, Florida International University, 33199 Miami, FL, USA; 2 Pulmonary & Critical Care Medicine, Miller School of Medicine, University of Miami, 33136 Miami, FL, USA; 3 Human and Molecular Genetics, Herbert Wertheim College of Medicine, and Biomolecular Sciences Institute, Florida International University, 33199 Miami, FL, USA; 4 Dept. of Computational Medicine and Bioinformatics, College of Medicine, University of Michigan, 48109 Ann Arbor, MI, USA

**Keywords:** microbiome, co-occurrence networks, bacterial clubs, rival clubs, club leader

## Abstract

**Background:**

It is well understood that distinct communities of bacteria are present at different sites of the body, and that changes in the structure of these communities have strong implications for human health. Yet, challenges remain in understanding the complex interconnections between the bacterial taxa within these microbial communities and how they change during the progression of diseases. Many recent studies attempt to analyze the human microbiome using traditional ecological measures and cataloging differences in bacterial community membership. In this paper, we show how to push metagenomic analyses beyond mundane questions related to the bacterial taxonomic profiles that differentiate one sample from another.

**Methods:**

We develop tools and techniques that help us to investigate the nature of social interactions in microbial communities, and demonstrate ways of compactly capturing extensive information about these networks and visually conveying them in an effective manner. We define the concept of bacterial "social clubs", which are groups of taxa that tend to appear together in many samples. More importantly, we define the concept of "rival clubs", entire groups that tend to avoid occurring together in many samples. We show how to efficiently compute social clubs and rival clubs and demonstrate their utility with the help of examples including a smokers' dataset and a dataset from the Human Microbiome Project (HMP).

**Results:**

The tools developed provide a framework for analyzing relationships between bacterial taxa modeled as bacterial co-occurrence networks. The computational techniques also provide a framework for identifying clubs and rival clubs and for studying differences in the microbiomes (and their interactions) of two or more collections of samples.

**Conclusions:**

Microbial relationships are similar to those found in social networks. In this work, we assume that strong (positive or negative) tendencies to co-occur or co-infect is likely to have biological, physiological, or ecological significance, possibly as a result of cooperation or competition. As a consequence of the analysis, a variety of biological interpretations are conjectured. In the human microbiome context, the pattern of strength of interactions between bacterial taxa is unique to body site.

## Introduction

Complex, heterogeneous, interacting microbial communities reside in a variety of niches, including those within the human body and other host organisms [1]. The Human Microbiome Project (HMP) focuses on using metagenomics approaches to study microbial communities that inhabit the human body [2,3]. In healthy human beings, bacterial communities play such critical roles as digestion of food, synthesis of essential vitamins, and inducing the immune system to create antibodies. HMP studies have revealed that diseases and disorders are strongly correlated with changes in microbial community profiles [4-6]. These studies have also demonstrated that microbial community structure in five niches of the human body (gut, mouth, airways, urogenital, and skin) are quite distinct, and appear to transcend gender, age, and ethnicity [7].

In recent efforts, microbiome studies have involved extracting microbial DNA from a sample followed by next generation sequencing. Classifying the reads helps to generate the profiles (either taxonomic or functional) of these microbial samples. Analysis of these profiles can shed light on the microbial communities - their commonalities and their differences - in the samples being investigated.

Still, metagenomic studies have to go further and dig deeper to uncover interesting features of microbial communities. The next set of promising investigations must focus on understanding the structure of microbial communities and their interactions within the context of an environmental niche. One of the greatest challenges in understanding human health is uncovering the large number of complex interactions that occur within the microbial community, and between the community and the human host. There is a great need to interpret the results in a way that is useful to both research scientists and clinicians. Studying the structure of microbial communities will shed light on the nature of bacterial "social networks" and their consequences.

Sequencing data is typically clustered into operational taxonomic units (OTUs) based on similarity, with taxonomic identity then assigned to each OTU. This is useful for describing the phylogenetic nature of a microbiome, but such a snapshot does little for describing interactions between community members. We claim that viewing a metagenome as a social network of OTUs could lead to greater insights into what is a normal community, and how it can be disrupted by external changes or invasions by non-members. In this paper, we describe methods for constructing these networks, and define structures that could be of key importance for discovering the interactions that occur. Our goal is not to describe the results of specific experiments, but to suggest ways of finding these structures premised on the idea that they may have biological importance. We hope to begin defining a new vocabulary for microbial social networks, one which borrows ideas from traditional statistics, machine learning, and graph theory, but which is better suited to the idiosyncrasies of the microbial communities themselves and the language previously used to describe them.

### Reads, OTUs, and abundance matrices

Sequencing technologies are constantly improving, thus increasing the confidence in the quality of the reads that are generated. A major source of error remains the process of classification of the reads, whether it is in terms of taxonomy or functional annotations [8]. Any classification process is limited in its accuracy by the quality of the reference databases available [9]. The best known marker gene remains the 16S rRNA gene. The Ribosomal Database Project (RDP) has cataloged nearly 3 million 16S sequences by bacterial taxa [10]. The size and coverage of this and other databases dictate the limits on the ability of all classification methods.

A second major limitation is the inherent ability of a marker gene such as 16S rRNA to distinguish bacterial taxa and resolve taxonomic identity. Our current understanding is that the gene for 16S rRNA is present in every bacterial genome. It contains a mixture of highly conserved and hypervariable regions, the former making it an easy target for amplification and the latter the reason for its usefulness in mapping reads to taxa [11]. However, in many cases, the rRNA gene has little or no variability within strains, species, or even bacterial genera [12]. This implies that the results can only provide profiles painted with "broad brush strokes". This can be problematic for our purposes, since members of the same genera can behave very differently. For example, *Campylobacter hominis *is considered a member of the normal flora of the gut, whereas *C. jejuni *is known to be pathogenic [13]. Furthermore, closely related bacterial strains are often competitors for the same environmental niches [14]. Thus even though it may be desirable to differentiate between strains in order to better understand the dynamics of the communities being studied, it may not be possible to do so with the marker gene used.

The limited resolution of amplicon-based methods does not mean that reads cannot be intelligently assigned to distinct groups. One way to get around this limitation is achieved by clustering the reads into OTUs based on sequence similarity, and then classifying the clusters in the best possible manner. Even though many clusters may be classified as being part of the same taxon, the dissimilarity between their sequences would suggest that the clusters represented different taxonomic groups at a lower level [15]. This typically results in several OTUs belonging to the same taxon, with each OTU being roughly analogous to a strain or species at or above 97% sequence similarity. In many cases, where the system is unable to classify at a certain taxonomic level, it is still capable of classifying at the next higher taxonomic level (i.e., not at the genus level but at the family level).

While our discussions in this paper are mostly confined to the 16S rRNA marker gene, the tools and techniques related to community interactions presented here are independent of how the community profiles are generated. They could just as easily be applied to functional annotations instead of taxonomic classifications. As an example, tools such as PICRUSt [16] exist for prediction of functional components in a microbiome. In the networks described below, substituting taxonomic identity with predicted function for each node could produce insights into the social dependencies required for a community to operate normally, and which functions have the greatest influences in different niches.

### Analyzing the abundance matrix

The data that results from the OTU-based analysis and classification is a simple *abundance *matrix *M *whose rows correspond to subjects or samples and columns correspond to bacterial taxa or OTUs present in that sample. Each entry in the matrix quantifies the abundance of that OTU in a specific sample. The rows of the matrix are assumed to be partitioned into groups that represent classes of subjects being studied. For example, the groups may correspond to normal versus diseased subjects, or smokers versus non-smokers.

There are a number of standard ecological measures that can be employed to gain some insight into the complexity of the environment being studied, including estimating the richness and diversity of the community. Richness simply indicates (an estimate of) the number of different OTUs present in a sample given that it is not possible to make an exact count [17]. Diversity goes a step further and considers how these different OTUs are distributed. A diversity estimate indicates the variability in the number of members within those OTUs [18]. If each OTU has a similar number of members, then the diversity of the community is relatively high, whereas if a few OTUs make up the bulk of all the individuals present, then diversity is considered very low.

Richness and diversity measures may shed light on the complexity of a community. They are believed to be useful when contrasting communities, such as those housed by different human subjects at the same body site, or at different body sites in the same subject [19]. However, efforts in our lab have not supported this claim (unpublished results). Richness and diversity measures provide precious little information about specific differences between samples because the questions they answer are very broad and general. Something obvious and more interesting is to ask which bacterial taxa are prominent in one sample as compared to another. Many tools (for example, see [20]) have been presented for the purpose of identifying statistically significant differences in the abundances of OTUs, but tools which can help us intuitively understand those differences are still needed for helping us generate better targeted questions.

## Results

Visualization tools provide meaningful qualitative approaches for the analyses of community structure, and they can be complemented by more analytical tools for comprehensive quantitative analyses. We first discuss our visualization results and then discuss the other analytical tools employed in this paper. We apply this suite of network-based tools for the analyses of microbiome data as described below.

### Bacterial co-occurrence networks

**Figure 1:**
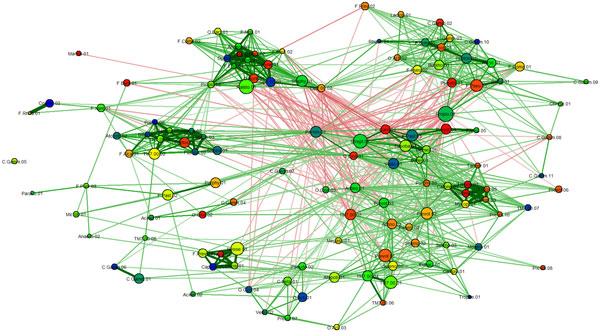
**Co-occurrence network for bacterial OTUs**. Edges representing weak correlations (absolute value less than 0.2) are not shown in order to make the visualization cleaner and less cluttered. Node coloring is such that redder colors indicate OTUs that are more differentiable in terms of their abundance between two groups (e.g., former smokers and non-smokers)

**Figure 2:**
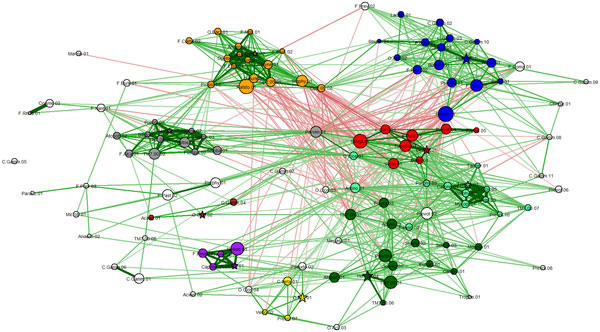
**Co-occurrence network for bacterial OTUs**. All features are as in Fig. 1, except for node coloring. Different node colors indicate different bacterial "social" clubs (clusters), automatically generated using the Markov clustering method. Clubs are indicated by nodes of the same color. Groups with fewer than 3 nodes are not considered clubs. Uncolored vertices are not part of any club.

*Bacterial co-occurrence network *diagrams were generated using the qgraph package for R [21] (Figures 1 and 2). These networks were visualized with the aid of the *Fruchterman-Reingold algorithm*, a force-directed method of arranging nodes based on their interactions.

### Bacterial clubs

Since the Fruchterman-Reingold method tends to locate correlated OTUs close to each other, visual identification of *bacterial clubs*, i.e., clusters of co-occurring bacterial taxa, is often obvious in many networks. For example, a visual inspection of Figure 2 suggests many distinguishable clubs, characterized by a set of closely located nodes connected predominantly by thick green edges.

**Figure 3:**
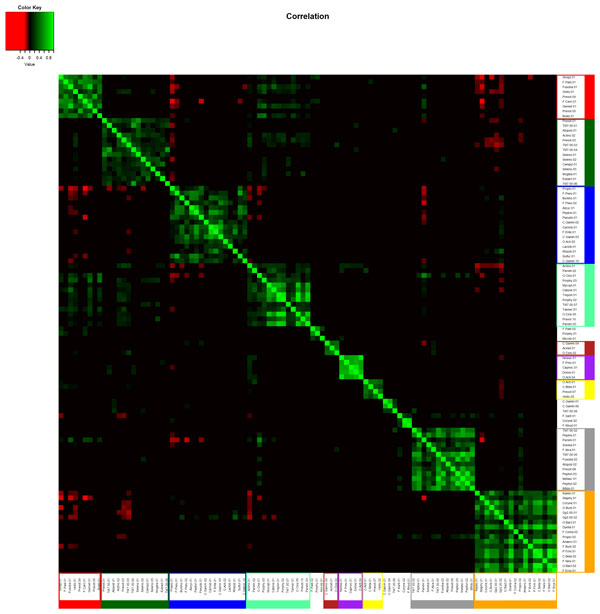
**Heat map showing "clubs" generated for the data set from Figures 1 and 2**. OTU labels are color-coded to match the color scheme for Figure 2.

**Figure 4:**
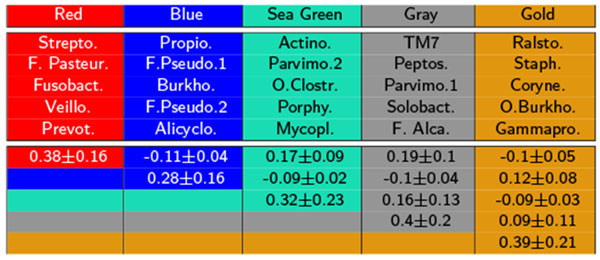
**The five most abundant OTU members in five of the clubs (colored red, blue, sea green, gray, and gold in Figure 2) with measures of (mean±SD) inter- and intra-correlations between clubs using only significant correlations**. Names of bacterial taxa are abbreviated for convenience. Two (marked) pairs of rival clubs found (by our algorithm) in the network from Figure 2.

It is important not to rely solely on visual aids to identify bacterial clubs. Using Markov clustering, we were able to confirm that the visually observed clusters in the diagram can indeed be automatically identified. Our experiments show that the Markov method does a reasonable job of clustering bacterial co-occurrence networks and finding agreement with the more qualitative network diagram approach. This is shown in the heat map (Figure 3) and in the marked network graph (Figure 2). Note that the scheme for coloring nodes in the network shown in Figure 1 is different from that used in Figure 2. In Figure 2, membership in a bacterial club is denoted by node color. Furthermore, we note that the results in Figure 2 contain the clubs that were visually identified in Figure 1, but the node color is used to represent differential expression between active smokers and never smokers. The five most abundant bacterial taxa in the five largest clubs, which resulted from the Markov clustering of the network shown in Figure 2, are listed in the form of a table in Figure 4.

The Markov clustering method [22] also computes "attractor" nodes. They are marked with a star in Figure 2. These "club leader" nodes are not easily inferred visually, and are one of the advantages of using the Markov clustering algorithm for unsupervised discovery of clubs.

### Rival bacterial clubs

*Rival bacterial clubs *were initially observed by visual inspection. For example, the club with gold nodes (toward the upper left) in Figure 2 has many red edges emanating from it.

**Figure 5:**
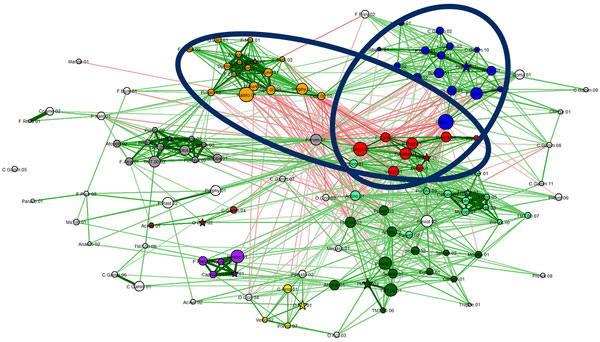
**Two (marked) pairs of rival clubs found (by our algorithm) in the network from Figure 2**.

The two-phase algorithm described in the *Methods *section for computing rival bacterial clubs was employed on all our bacterial co-occurrence networks. As stated above, the results computed by this method agree with what was identified by visual inspection. In Figure 2, the clubs colored green and gold form "rival clubs". This pair of rival clubs is marked in Figure 5. For example, the average correlation value between members of the red club is 0.38 ± 0.16. The average correlation value between members of the gold club is 0.39 ± 0.21. The average correlation value between members of the green club and the gold club is −0.1 ± 0.05. A second rivalry is also shown to exist between the red and blue clubs.

### Smokers' microbiome

**Figure 6:**
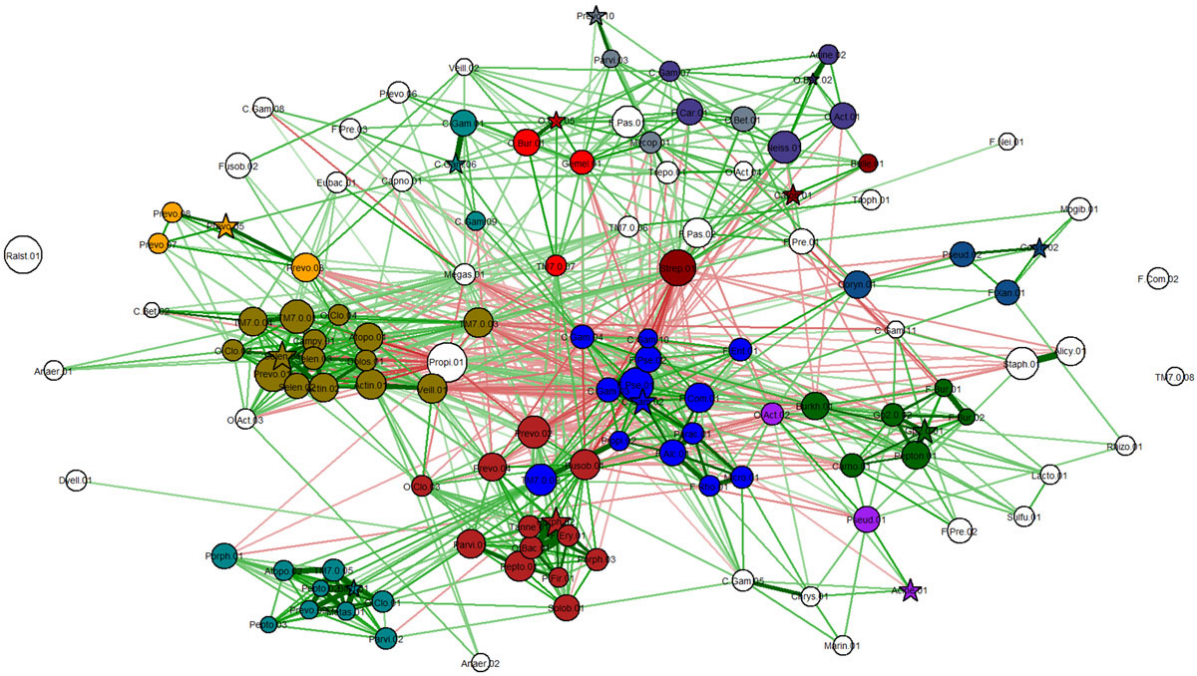
**Bacterial co-occurrence network with clubs for smokers**.

We applied our techniques to data generated from a project analyzing the airways microbiome of 22 smokers and 24 former smokers. The results from applying the network-based analysis presented in this paper on the data from the 24 former smokers are shown in Figures 1 and 2. The results from the 22 samples collected from smokers are shown in Figure 6.

### HMP datasets

**Figure 7:**
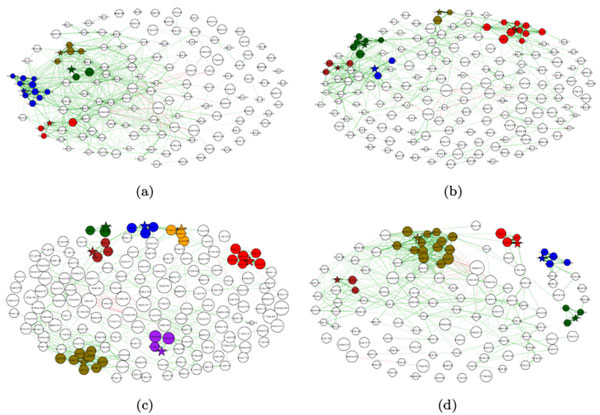
**Bacterial co-occurrence networks for HMP data sets: (a) Supragingival; (b) Subgingival plaque; (ac) Saliva and (d) Buccal mucosa**.

**Figure 8:**
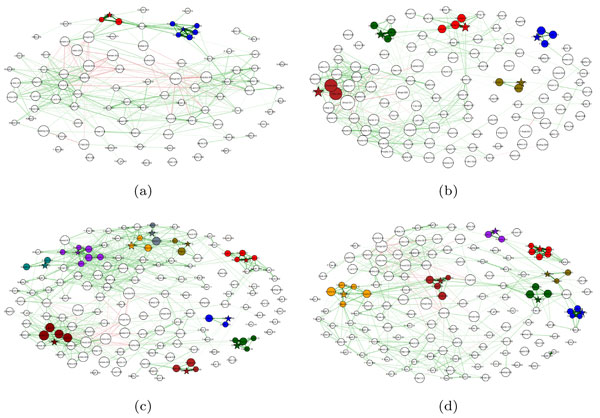
**Bacterial co-occurrence networks for HMP data sets: (a) Tongue Dorsum and (b) Hard Palate (c) Palatine Tonsils and (d) Throat**.

The bacterial co-occurrence networks for the HMP data sets for eight different body sites are shown in Figures 7 (a)-(d) and 8 (a)-(d).

## Discussion

Bacterial co-occurrence networks show which bacterial taxa co-infect subjects of the same type, and end up in the same niche to form microbial communities. These network diagrams are a natural way to visualize such relationships since the nodes represent OTUs and edges represent co-occurrence relationships. They contain a large amount of information in a compact way. The challenge is in analyzing and interpreting such network diagrams.

The edges in the network diagram in Figure 1 and 2 indicate the tendency of the OTUs to co-occur in samples. These relationships are similar to those found in social networks [23]. Assuming that strong (positive or negative) tendencies to co-occur must have biological, physiological, or ecological significance, we extrapolate these edges as being indicative of the strength of their relationships. We assume that strong relationships are a result of cooperation or competition. Cooperation and competition between bacteria has been well studied in the field of bacterial ecology.

It is therefore natural to ask whether these network diagrams reveal interactions between bacterial taxa. In this context, it makes sense to ask if there are "clusters" in the network graphs and if these clusters are different for different groups of samples. *Cluster analysis *refers to a collection of methods that identify "natural" groups within a class of entities. We will refer to these clusters as social *clubs*, or simply clubs. We informally define a *club *to be a group of bacterial OTUs with strong positive correlations between each other.

A visual approach to finding clusters was aided by the *Fruchterman-Reingold algorithm*, which places strongly positively correlated OTUs fairly close to each other, causing them to form visually identifiable clusters. Clusters of strongly correlated bacterial taxa were immediately obvious in the co-occurrence networks that were produced by the layout algorithm. However, we also have negative correlations present in the data. The Fruchterman-Reingold algorithm also tends to co-locate OTUs that are strongly negatively correlated. Thus it may be possible to identify clusters with strong correlations (either positive or negative). One note of caution is that if two nodes are located close to each other, it does not imply that they are strongly correlated to each other, because the lack of correlation is not a strong "repulsive" force. Thus there is no clear delineation between groups of positively and negatively correlated OTUs in the co-occurrence networks produced by the Fruchterman-Reingold algorithm.

As mentioned above, the Fruchterman-Reingold algorithm is merely used as a visualization tool to observe the bacterial co-occurrence networks, which have interesting clubs. Of course, it is much more useful to automate the process of finding meaningful clubs. Considerable research exists on the problem of finding such clusters (i.e., clubs) in a (weighted) network. Existing methods include spectral clustering [24], edge-based agglomerative or divisive methods [25], multi-level graph partitioning [26], algorithms based on Min-cut [27], Markov clustering [22,28], and much more [29-33]. The problem is also similar to that of identifying high-density subgraphs [34] and can be computed (with minor modifications) using an algorithm by Hartuv and Shamir [35,36] or the one by Hüffner et al. [34]. All the above methods have their strengths and weaknesses, but the Markov clustering approach was chosen for our work because of its previous success with biological data sets [37]. Note that the limitations of Markov clustering include a lack of proof of convergence, limited ability to deal with graphs of large diameters, lack of robustness, and the fact that some of the parameters are set arbitrarily [22,28].

Our experiments show that the bacterial clubs identified by the Markov clustering method are consistent with the visual clusters observed using the Fruchterman-Reingold method. The bacterial clubs can be seen in the heat map (Figure 3) and in the marked network graph (Figure 2).

Clubs with predominantly positive correlations are likely to indicate "cooperation" between the members of the group. The interacting OTUs may represent taxa that depend on or complement each other in a given environment and could indicate a core group of functions needed to thrive. Negative correlations between bacterial taxa suggest "competition" between members of the group. While large groups of bacterial taxa with strong positive correlations are likely to exist, large groups of bacteria with strong negative correlations are not likely to exist since the definition of correlation does not quite permit it. However, large groups of weakly negative correlations may be found.

A substantially more interesting structure in the network diagrams is the "competing groups" of bacterial OTUs. We informally define a pair of *rival clubs *to be a pair of clubs such that members of one club have negative correlations with the members of the "rival" club. Rival clubs are likely to indicate groups that are either clamoring for the same scarce resources in the given environment or producing byproducts toxic to each other.

*Rival bacterial clubs *can often be easily observed by visual inspection. For example, the club with blue nodes in Figure 2 has many red edges emanating from it. The strong negative correlations with other bacterial taxa suggests an important incompatibility between this bacterial club and its neighbors.

The Markov clustering method [22], which was used to identify bacterial clubs also computes "attractor" nodes. We asked the question whether the concept of an attractor could have any biological significance. Conjecturing a potential leadership role for these bacterial taxa, we refer to these attractor nodes as *club leaders*. It is an OTU that has the most dominant set of correlation values with the other OTUs, suggesting that it may have a critical role to play in the community. One biological interpretation of club leaders is that they could be providing some essential resource to the club members. We add a note of caution that the conjectures about club leaders do not have any supporting evidence as yet and should be considered as speculatory. The attractor nodes are not easy to infer visually. The attractors or club leaders are marked with a star in Figure 2.

Note that in practice, there could be times when the network visualization and Markov clustering methods result in non-negligible differences due to limitations in the sensitivity of their underlying algorithms. We propose using both methods, the visual tool and the clustering tool, to complement and validate each other. We would recommend close manual curation of the data in the event of very large discrepancies.

### Smokers' microbiome

The techniques developed in this paper were applied to many data sets. First they were applied to the smokers' data set. Broadly speaking, the results showed greater rivalries in the microbial communities of active smokers than those in the former smokers. Even though the actual OTUs may represent very different taxa, the club with blue colored nodes in Figure 2 appears to have many common taxa with the club colored blue in Figure 6. The blue clubs in Figure 6 and 2 have a fairly high number of red edges (negative correlations), which could suggest clubs with general antagonistic behavior that is common in the lungs of all types of people.

### HMP datasets

Bacterial clubs were also found in data from the Human Microbiome Project, although they tended to be fairly small when compared to the smokers' data set. The subgingival plaque microbiome showed less interactions between the clubs than the supragingival plaque microbiome. The saliva microbiome showed less coherence than the buccal mucosa microbiome. There were more positive interactions between the bacterial clubs in the hard palate microbiome than in the tongue dorsum microbiome. The tongue dorsum microbiome showed some weak rivalry between the clubs. Finally, the throat microbiome showed a very strong club with an average positive correlation of 0.97, suggesting that the throat has at least part of its microbiome consisting of a very stable group of OTUs.

Others have begun using similar methods to better understand interactions in the human microbiome. Indeed, Faust [38] has also attempted to capture complex forms of ecological interactions using co-occurrence networks of the type described here. A comparison of those results in the oral cavity with our diagrams are in strong agreement. Likewise, application of our methods to data from the buccal mucosa find similar associations as in [39]. However, our methods add to the above results by suggesting cohesiveness between specific community members and apparent antagonism between others. Noting the associations is valuable, but our methods can help generate the right questions to ultimately disentangle the interactions that can be inferred from these structures.

In summary, we note that different body sites contain clear differences in the clusters of OTUs present, supporting the hypothesis that the pattern of strengths of interactions between bacterial taxa is differentiable when comparing different body sites. Even if the same OTUs are present at different sites, their behavior is not necessarily the same. This could be due to differences in the environment, but could also be in response to differences in the presence or absence of other OTUs and their abundance levels. Further work is required to understand the meaning of these interactions and why the differences exist, but perhaps it is enough to note that the structures of these networks differ. Our techniques provide a framework for picking out those differences for further study.

## Methods

### Methods employed

*Bacterial co-occurrence networks *are networks where the nodes represent OTUs and the edges represent co-occurrence relationships. All network diagrams were generated using the qgraph package for R [21]. The package provides flexible ways of drawing and coloring nodes and edges. This flexibility allowed for overloading the network diagrams with many additional pieces of information. The following is a complete description of the networks we created along with all the information associated with its components.

1. Each node of the network corresponds to an OTU whose presence has been detected in one of the study samples.

2. An edge connecting two nodes is used to represent the "co-occurrence" *relationship between two bacterial taxa*. The strength of the co-occurrence is computed as a correlation coefficient and is reflected in the *thickness of the edge*. Correlation coefficients are computed in our experiments using the traditional Pearson Correlation Coefficient.

3. Significance of each correlation was evaluated using the corr.test() function in R. The false discovery rate was estimated using the p.adjust() function and method "BH". Because the force directed layout method of the Fruchterman-Reingold algorithm depends on the strength of the correlations between nodes, and because spurious correlations can influence the placement of nodes and the appearance of clusters (or "clubs"), a method of neutralizing the effects of those spurious correlations was implemented. All correlations with an FDR-adjusted significance above 0.25 were set to a value of zero for the construction of network diagrams, as well as in the Markov clustering step. The threshold of significance was set to a high value to account for influences which, while not significant at an alpha level of 0.05, are still likely to have some effect on the interactions between OTUs.

4. The *color of the edge *is used to indicate whether the correlation is positive or negative. A green edge is used for a positive correlation, a red edge for a negative correlation.

5. Since abundance is an obvious quantitative measure for any OTU detected to be present in a given subject, the *size of a node *is representative of the average abundance across all samples. After normalizing the abundance values, the resulting *relative abundance *or *normalized abundance *values are used. Since abundance values have a wide range spanning several orders of magnitude, the normalized values were log-transformed. Correlations were calculated based on these normalized and log-transformed values.

6. A *thresholding *process was used to discard all OTUs whose abundance was not "sufficiently high". This helped to focus the process on fewer and more relevant OTUs. In our work, if the total number of reads was less than 100, or the OTU was present in fewer than 20% of the members of a study group, then the OTU was discarded.

7. There are many network drawing strategies that have been developed in the literature. We used the Fruchterman-Reingold method to draw the network [40]. This method tends to locate nodes connected by edges of large weight closer to each other than nodes connected by edges of small weight. Additional details on this method can be seen below in the Section titled "Fruchterman-Reingold algorithm".

8. If a specific OTU is more abundant in one set of samples as compared to the other, then it is considered to be of special interest. The color of the nodes is indicative of how differentially abundant a given OTU is for the given groups of samples. If the data contains only two groups of samples, a simple *t*-test can provide quantitative evidence indicating the extent of differentiability for each OTU. Nodes can then be colored according to a heat scale. The less significant the difference in abundance between two groups, the cooler (bluer) the color. The greater the significance in the difference, the hotter (redder) the color of the node. For multiple groups, other sophisticated methods can be used.

9. Each OTU is labeled with the best taxon to which it maps. As mentioned before, multiple OTUs may be mapped to the same taxon. In order to distinguish between them, an arbitrary number is appended to the label.

10 Validation of networks and clusters was performed by first constructing a correlation network using the SparCC approach [39] and then repeating all of the analytical steps in the workflow.

11. For the smoker data sets, runtimes for construction of the network with 126 nodes and 1,581 edges and the Markov clusters were 3.47 seconds and 2.53 seconds respectively, running 64-bit Windows 7 Professional on a 3.6 GHz Dell with an Intel(R) Core(TM) I7-4790 processor with 16 GB RAM.

#### Fruchterman-Reingold algorithm

The Fruchterman-Reingold algorithm produces force-directed layouts of networks [40]. Given a set of nodes with weighted edges connecting them, the algorithm works as follows. Imagine that a spring exists between every pair of nodes. The strength of the springs varies depending on the weight of the edge connecting them. Initially, each node is placed at an arbitrary position in space, and the overall energy of the system due to the pull of the springs is calculated. Two nodes with a relatively "strong" edge connecting them will tend to attract each other, but there may be many other interactions acting to pull them in different directions. The position of the nodes is then readjusted in a stepwise manner according to these combinations of forces, and the overall energy of the system is again calculated. This process is repeated until the layout with the minimal overall energy is found. The position of each node is thus dependent on the strength of its edges with all other nodes.

#### Markov Clustering

The Markov clustering approach [37] was used for computing bacterial clubs in the bacterial co-occurrence networks. We describe this algorithm briefly. For more details, see [22]. It exploits the idea that a random walk in the network would have the property that once it enters a "dense cluster" it would end up getting trapped in it until the entities in the cluster have been visited many times. The actual algorithm simulates a flow (instead of a random walk) and then by strategically increasing or decreasing the flow on select edges, it achieves decreasing flow across clusters while keeping all the flow circulating within clusters. The process of strengthening or weakening flows in select links is referred to as "inflation" and can be parameterized to obtain clusterings of varying granularity (i.e., stricter or laxer clusterings).

### Computing clubs

#### Computing rival clubs

As mentioned before, clubs can be automatically identified in a network using clustering techniques. On the other hand, rival clubs can be identified in a network using biclustering techniques. Many approximation algorithms exist in the literature for this problem (for example, see [41,42]). Many clustering and biclustering algorithms also provide statistical significance information [42]. Unfortunately, most of these methods are not geared for dealing with correlation networks (i.e., graphs with positive and negative weights).

We employed the following two-phase algorithm. We first let the correlation values be replaced by their absolute values, making all correlations to be positive. Using a basic clustering algorithm, we generated super-clubs, which ensured strong correlations within each club, but ignored the sign of the correlations. In phase 2, the basic Markov clustering algorithm was applied to each club computed in the first phase, but this time with all negative correlation edges removed. Note that since we were performing Markov clustering on the super-clubs, any pair of clubs coming from the same super-cluster must have very few positive correlations between each other, and consequently must have many negative correlations. This method was effective and efficient in finding rival clubs.

#### Identifying Club Leaders

The work of [22] has shown the existence of a special node in each Markov cluster computed by the algorithm where the flow seems to "terminate" after many iterations. As the Markov clustering process progresses, the simulated flow ends up getting stronger and stronger to one single vertex in each cluster, which is referred to as its *attractor *[22]. These attractor nodes are referred to as *club leaders*. The Markov cluster algorithm is able to perform this task effectively and efficiently.

### Dataset processing

#### The smoker's microbiome

We applied our techniques to data generated from a project analyzing the airways microbiome of 22 active (15 male, 7 female, mean age 52.7) and 24 former smokers (13 male, 11 female, mean age 55.4). DNA was extracted from lung bronchoalveolar lavage of all the subjects. This was followed by a PCR amplification of the V6-V8 hypervariable region of the 16S rRNA operon using custom-designed degenerate primers [43,44], followed by next generation sequencing. The sequencing data was then subjected to a standard metagenomics pipeline and an abundance profile for each of the OTUs was created for each sample. The network-based analysis presented in this paper was then applied to the resulting data. The results from the data on 24 former smokers was shown in Figures 1 and 2. The results from the smokers are shown in Figure 6.

#### HMP dataset

Raw 16S data was downloaded from the Human Microbiome Project's (HMP) DACC Data Portal and run through the same analysis pipeline. Samples came from eight oral and airways body sites, including saliva, buccal mucosa, tongue dorsum, hard palate, subgingival plaque, supragingival plaque, palatine tonsils, and throat. Details of data collection methods are available at http://hmpdacc.org. Each of the eight data sets had an average of over 150 subjects sampled, making a total of about 1200 subjects. The analysis was limited to samples from the host subject's first visit.

## Conclusion

In this paper, we propose techniques to study bacterial co-occurrence networks to infer potential interactions between the bacterial taxa present in a microbial community. We introduce new concepts called "clubs", "club leaders", and "rival clubs" that can assist in identifying cooperating and competing groups of bacterial taxa. These techniques are timely as metagenomics studies attempt to tease out increasingly complex relationships between the members of microbial communities.

## Competing interests

The authors declare that they have no competing interests.

## Authors' contributions

MF and JDR performed all the computational work and analyses. MF and GN wrote the paper. All authors reviewed the paper.

## Dedication

We dedicate this paper to the memory of our colleague, Melita Jaric.

## Declarations

Publication cost of this article was borne by GN.

This article has been published as part of *BMC Genomics *Volume 16 Supplement 11, 2015: Selected articles from the Fourth IEEE International Conference on Computational Advances in Bio and medical Sciences (ICCABS 2014): Genomics. The full contents of the supplement are available online at http://www.biomedcentral.com/bmcgenomics/supplements/16/S11.
